# Agropastoral Mobility and Rangelands Multiple Uses in the Miombo Frontier Ecozone of Tabora Region, Western Tanzania

**DOI:** 10.1155/2017/5835108

**Published:** 2017-10-30

**Authors:** George Felix Masanja

**Affiliations:** Department of Geography, St. Augustine University of Tanzania, Mwanza, Tanzania

## Abstract

This study aimed to examine the argument of environmental resource-use conflict as the primary cause of crop farmers and agropastoralists conflicts in Tabora Region, Tanzania. It explored the multiple interdependent phenomena that affect livelihoods relationships between crop farmers and agropastoralists and the nature of their continuing conflicts over the ecozonal resources. A primary dataset of the two groups' conflicts was used. An ex post facto and multistage sampling design was adopted. A total of 252 respondents were interviewed in three separate villages drawn from agroecological zones fringing the miombo woodland where such tensions are high. Data were analyzed using logistic regression. Results indicate that education (*β* = −1.215, .297; *p* = .050), household size (*β* = .958, 2.607; *p* = .017), herd size (*β* = 4.276, 7.197; *p* = 0.001), farm size (*β* = −1.734, .048; *p* = .176), the police (*β* = −.912, 4.582; *p* = .043), and village leaders (*β* = −.122, .885; *p* = .012) were the most potent predictors of causes of conflicts. The study found no support for demographic variables, like age, sex, marital status, income, duration of residence, and distance to resource base. The study recommends population growth control and strengthening of local institutions and recommends local communities to sustain management of natural resources base in the area.

## 1. Introduction

The communal rangelands in Africa are characterized by conflicts related to environmental resource uses, of which Tanzania is not an exception. Given the fact that the struggle for available land resources brings conflicts between the crop farmers and agropastoralists [1–3] there is every need to verify how both parties perceive the impact of such clashes on crop production and cattle rearing. This assertion is very critical because the production of crops and that of livestock under subsistence systems are not mutually exclusive due to their competition for the available land resources for their survival. The rapid increase in human and livestock population in Tanzania has brought up a requirement of land for grazing and production of crops which, inevitably, has led to land-use conflicts. Most agropastoralists in Tanzania are always moving. They make a response to the variable and unpredictable environments by moving across to access the available forage resources [4]. Presently, both small-scale farmers and agropastoralists are scattered all over the country with no demarcation between grazing areas and cropping zones. Worse still, crop farmers plant without fencing and the animals graze on open grasslands without being confined to specified grazing areas too. The encroachments are on both sides, and the problem is further compounded by both crop farmers and agropastoralists moving close to points of water during dry seasons [5, 6]. This contention brings both parties into contact with a consequent escalation of conflicts. The rapid population dynamics of both livestock and human populations have triggered herders' migration that has, consequently, led to resources-use conflict by the convergence of pastoralists and farmers [7].

The setting up of communal enclosures in most African lands suitable for grazing livestock has been an important coping strategy in response to decreasing grazing lands and diminishing patterns of livestock mobility due to human population increase [8, 9]. Despite the important role played by enclosures in the restoration of degraded rangelands, [10, 11] indicate that enclosing communal rangelands may bring up social conflicts and cause degradation of grazing land, rather than contributing to rehabilitation. Likewise, the government efforts to establish village grazing areas where livestock keepers can be confined has also left undesirable ends. Thus, lack of sufficient pasture and water supplies has motivated herders to move around searching for such resources elsewhere [1].

Following these circumstances, the late 1970s saw the beginning of the large-scale exodus of Sukuma agropastoralists (whose livelihoods integrate farming and herding at varying ratios), southwards from the densely populated cleared steppe areas of Mwanza and Shinyanga Regions into the miombo woodlands of Tabora, Sikonge, and Urambo districts in response to the changing environmental and climatic factors followed by drought cycles that have become longer and more frequent [12, 13].

According to [14] declining environmental conditions (e.g., available land, soil fertility, and rainfall) often trigger decisions to out-migrate. Tabora Region currently has the fourth highest rural population growth rate of 2.9 percent in the nation [15]. The movement into the miombo woodlands was facilitated by the strip clearing along the lines of road and rail and the increase of clearing for Tobacco growing [16]. The beginning of this move was captured from the record of the presence of cattle on cleared patches of the miombo woodlands, as well as the empty settlements in Nzega District, abandoned due to overstocking [17]. The region had 2,133,090 (about 9.3 percent of the national herd) of which Tabora Urban, Uyui, Sikonge, and Urambo districts together had 1,043,848 livestock units [18], a figure growing more rapidly than the human population growth rate. The total figure had shot up to 3,353,766 by 2012 [19].

Although the regional population growth rates and average household sizes appear low and stable, they mask remarkable variations between wards and villages. Average household sizes have risen over time indicating that demographic trends at that level are more dynamic than demonstrated by the regional data. For example, the household sizes for rural districts of Tabora Region rose from 4.5 to 5.0 and 5.7 and 5.8 for 1967, 1978, 1988, and 2002, respectively. These figures deviate positively from the national average recorded for the same period at 4.4, 4.9, 5.2, and 4.9. Fertility levels are high in the region, with women capable of conceiving 6 or more pregnancies in a lifetime in some of the villages [20]. The region's average household size of 6.0 persons was the third highest rate in the country. This is exacerbated by an observed trend of declining age of first pregnancies. According to the 2012 national census, the region had a population of 2,291,623. For 2002–2012, the region's 2.9 percent average annual population growth rate was the ninth highest in the country.

**Figure 1:**
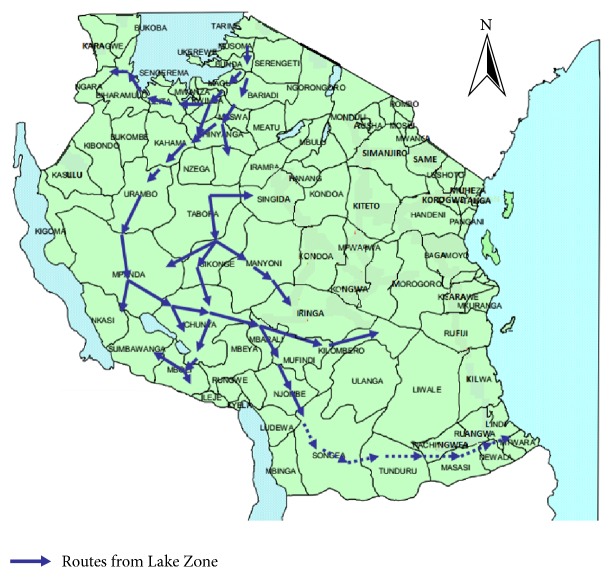
Southward agropastoral migration routes in Tanzania. Source: adapted and modified from Sendalo, 2009.

The incursion of agropastoralists from Shinyanga and Mwanza into the frontier areas of the miombo woodlands in Tabora Region (see Figure 1) has in the first place increased the number of large animals especially cattle, goats, sheep, and donkeys. This situation has promoted conflicts over the land and water resources and, sometimes, has led to violent clashes between these groups and host farmers. The agropastoralists have been attracted to move into the frontier forests of the miombo forests mainly due to the availability of fertile land for agriculture, pastureland, and ample water resources. However, their resettlement into these fragile environments has met stubborn resistance, not only from the locals but also from the government. The Wildlife Act Number 9 of 2009, the Ramsar Site Regulations and Requirements, and the Forestry Act of 2008, altogether, have a negative passion of human settlements and their economic activities within these protected areas.

Secondly, the settlement of these agropastoralists in their new areas has disrupted land-use patterns of the host farmers. As agropastoralists adapt their livelihoods practices to their changing circumstances, traditional rules facilitating cooperation between host farmers and incoming herders are becoming insufficient to manage increased competition over land and water. Relationships are increasingly becoming strained among the two parties following some crop farmers beginning to raise livestock too. These livelihoods change and with the breakdown of the collective village policy (Ujamaa) and the reduction of the land-use controls exerted during the socialist era [16] have altogether contributed to an expansion of the area of cultivated land, as well as an increment in the density of humans and animals on arable land. The continued immigration of people into the villages of Tabora Region has added to an already growing population, such that today, the key resources—fertile arable land, grazing, and water—are in increasingly short supply. In particular, wetland areas in the miombo rangelands have become a focus of in-migration and have heightened the competition for land and water, as farmers and herders, alike, converge on these centers of relatively high fertility and productivity [21]. Agriculture expanding into areas used for grazing on the one hand and agropastoralists frequently grazing their animals into crop farmers' farmlands, on the other hand, have been due to declining agropastoralists mobility. Escalating agropastoralists mobility and incursion together with the lack of effective land-use planning and uncontrolled population growth in the miombo woodland areas of Tabora Region have altogether contributed to the present state of affairs.

These conflicts have, in some areas, been very violent often ending in deaths, for example, in Isakamaliwa village, Igunga District, Tabora Region, in 2014. Other places where such cases have been reported include Magogo village in Igunga District, Kigwa B village in Uyui District, and Kaliua and Sikonge Districts. All these places are in Tabora Region [3, 22–24].

Many other studies have documented increasing conflict-induced frustrations experienced by these two groups within and outside Tabora Region [25–29]. However, according to [30], the linkage between agriculture and conflict has still not received sufficient analysis and discussion.

This study, therefore, explored the multiple interdependent phenomena that affect the relationships between agropastoralists and host crop farmers and the nature of their ongoing conflicts over natural resources. The specific objectives of the study were to identify the sociodemographic characteristics of the migrant agropastoralists and crop farmers in the study area and determine causes of conflicts between the migrant agropastoralists and crop farmers in the study area. Others include estimating the relationship between sociodemographic characteristics and causes of conflicts between migrant agropastoralists and crop farmers in the study area and identifying the policies and institutions involved in conflicts resolutions between agropastoralists and crop farmers in the study area as well as assessing the effectiveness of institutions involved in conflicts resolution between migrant agropastoralists and crop farmers in the study area.

Of more significance to this study is the fact that a body of knowledge relating the factors for the emergence and development of environmental resources conflicts and the way the affected groups of people utilize their frontier environment provides a basis for the understanding of spatial interactions among crop farmers and agropastoralists. Moreover, the study provides a basis for rational decision making on related aspects of residential neighborhoods among groups with different interests. This study strives to engage all actors in the understanding that social and environmental factors act in tandem to perpetuate the conflicts. Required improvements within settlements are also discussed. This contention means that the analysis of causes and effects of such conflicts will provide opportunities for intellectuals to link knowledge, the design of mediation measures, and practices which are the central concern of this study. Further, the study tries to reorient the thinking in mediation planning practices as a basis for developing reliable solutions. The study also brings to light the discussion on the need for desirable usability of communal land and its water resources within the existing frontier areas. Finally, the methods employed in this study provide a basis for analyzing other related environments in other contexts that have similar socioeconomic and cultural conditions to those prevailing in Tabora Region.

## 2. Theoretical Framework

There are several theoretical explanations of resource conflicts between multiple resource users. This study utilized the theory of the Tragedy of the Commons in conjunction with the Neo-Malthusian Theory of Environmental Scarcity and Conflict.

### 2.1. Theory of the Tragedy of the Commons

The economic theory of the Tragedy of the Commons propounded by [31] posits that when many users share an environmental resource, each one will try to reap the greatest benefit of it. The consequence will be overusing it and ignoring the group's collective interest, thus ultimately destroying the resource. In a review of Hardin's theory [32], it was argued that the expression Tragedy of the Commons has come to symbolize the degradation of the environment as a result of many individuals using scarce resources. The theory explains the “tragedy” by using the example of a grazing land which is accessible to all for use. This grazing land is used by herdsmen to graze their livestock. However, when these individuals overexploit a shared resource to the point that demand exceeds supply and the resource becomes scarce, the grazing land can no longer be sufficient to support the population. This commons crisis continues to stand out as an example of a great variety of existing resource problems such as scarcity of fresh water, arable land, and climate change. So, regarding Hardin's theory, the earth's atmosphere and the environment are the “common.” The “tragedy” is the damage done to the atmosphere that causes global warming, climate change, and environmental scarcity shared by all. Consequently, as these shared resources become limited, competition for them also increases among the users, thus leading to the rise of conflicts.

### 2.2. The Neo-Malthusian/Environmental Scarcity Theory

The Neo-Malthusian/Environmental Scarcity Theory predicts that the world population would soon overwhelm the resource base and lead to detrimental environmental destruction, unbearable hunger, and rampant conflicts. Homer-Dixon [33] is a scholar of the Neo-Malthusian position. He and other scholars, like [34, 35], presented an argument that resource scarcities can cause violent intraregion conflicts under unfavorable conditions [33]. He employed three hypotheses to link environmental changes with violent conflict. First, he posited that diminishing supplies of environmental resources, such as water and arable land, would trigger conflicts. The second hypothesis stated that sizable population movements, caused by environmental stress, would induce “group-identity” conflicts, especially ethnic clashes. The third hypothesis suggested that severe environmental scarcity would simultaneously increase economic deprivation and disrupt key social institutions, which in turn would cause “deprivation” conflicts, such as civil strife and insurgency [36].

These common and widely cited theoretical models have received scholarly attention since they also predict migration behavior from how environmental change differentially shapes household livelihoods [37, 38]. For example, inadequate farmland or insufficient rainfall causes some households to out-migrate to various destinations while others remain.

## 3. Materials and Methods

### 3.1. The Study Area

Tabora Region is located in western Tanzania on the central plateau, between latitude 3°–7° south and longitude 31°–34° east. The region is structured into seven districts with a total landmass area of about 76,151 km^2^ in extent, representing 9 percent of the land area of mainland Tanzania. Tabora is physically the largest of Tanzania's 30 regions. Just over two-thirds of this consists of Forest Reserves (34,698 km^2^) and Game Reserves (17,122 km^2^). Most of the region lies at 1,000–1,500 m above sea level, with two small areas in the northwest and southeast rising to 1,800 m above sea level. The topography over much of the area is flat to undulating with isolated hills and ridges representing outcrops of more resistant basement rocks.

The climate is warm with temperatures reaching their peak in September and October, just before the onset of the rainy season; mean annual temperature is around 23°C, with a slightly cooler period from May to July. Rainfall is seasonal, falling almost entirely between November and May. During the June to October dry season, occasional showers may occur. In the west of the region, average annual rainfall is over 1000 mm, decreasing to 700 mm or less in the east. Soils are 80–90 percent sand (ferric acrisol), with low organic carbon ranging between 0.4 and 0.8 percent. A total of 34,698 km^2^ is forest reserve, and 17,122 km^2^ is a game reserve.

Tabora Region lies in the unimodal upland plateau agroecological zone where agropastoralism and crop farming dominate the farming system. The natural vegetation in the area comprises the miombo woodlands (*Brachystegia, Julbernardia*) with mainly* Acacia* and* Combretum* spp. Land tenure is public and individual farmers have user rights to be allocated and to cultivate the land. Most of the population (93 percent) in the region depends on agricultural production and 80 percent of the regional economy is derived from agriculture.

As stated by the national census of 2012, the region had a population of 2,291,623 of which 78 percent live in the rural areas. For the intercensal period of 2002–2012, the region's 2.9 percent average annual population growth rate was the ninth highest in the country. During the last twenty years, the population of the area has increased rapidly.

### 3.2. Study Design

This study adopted a descriptive cross-sectional survey research design, ex post facto type, which provided a general framework for the collection of appropriate data that explores how land scarcity engenders conflicts between farmers and agropastoralists. It used an eclectic approach to both quantitative and qualitative methods to gather data and information on knowledge about land shortage, occurrences of conflicts, and policies about the use of land resources. The choice for this design rests on its ability to test cause and effect or correlation relationships. It is capable of seeking to reveal possible relationships by observing circumstances or condition and searching back in time for plausible contributing factors.

### 3.3. Population of the Study

The base population for this study consisted of the agropastoralists and crop farmers residing in the three study villages. The unit of analysis is a household.

### 3.4. Sample and Sampling Techniques

Multistage and stratified sampling techniques were used to select the respondents from the population of crop farmers and agropastoralists. Wards with perennial farmers-agropastoralists conflicts in each district were identified through interviews with region and district council officials during the pilot work in July 2016. These are as shown in Table 1.

**Figure 2:**
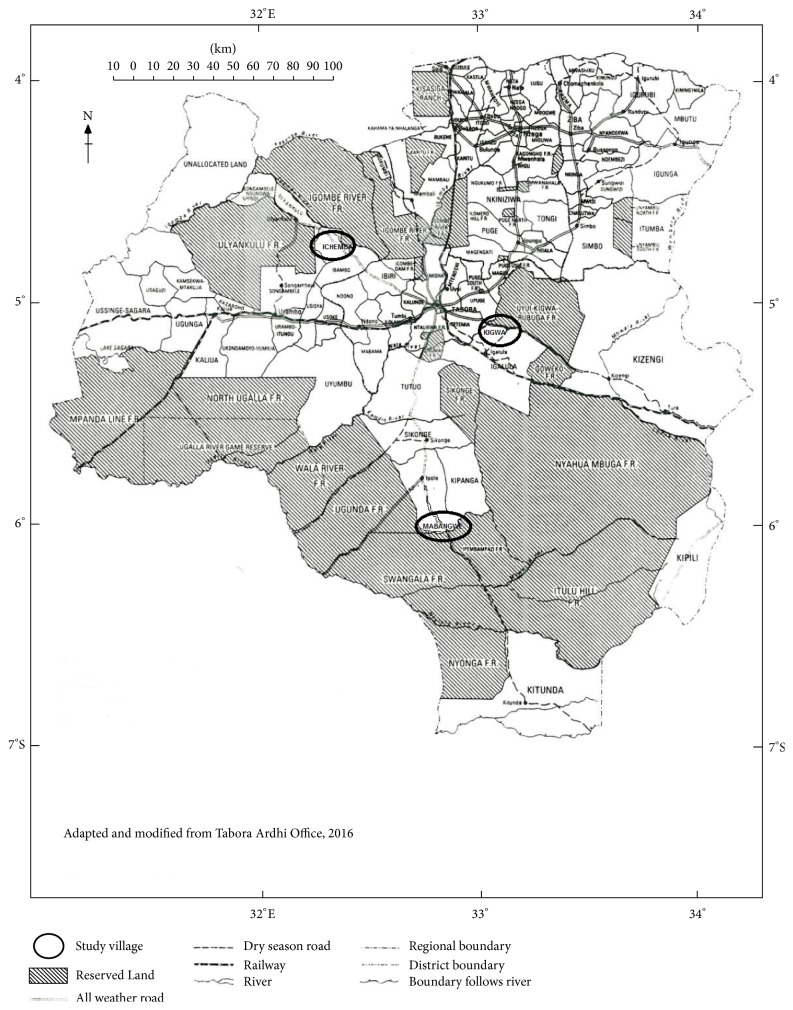
Map of Tabora Region showing study areas. Source: Tabora Lands Office, 2016.

In stage 1, fifty percent of these wards were randomly selected. The wards picked included Ichemba, Ngoywa, and Kigwa. The selection of these wards was also done using a stratified sampling procedure which took into consideration the three agroecological zones (cultivated land, upland vegetation, and wetland vegetation) covering Tabora Region. In stage 2, each selected ward produced one village which was purposively selected based on being a major in-migrant settlement and fresh reports of agropastoralist-farmer conflicts. In consultation with village council officials, the study villages (of which each had subvillages) containing substantial Sukuma populations were identified. These were Ichemba in Ichemba ward, Kigwa in Kigwa ward, and Mabangwe in Ngoywa ward. In stage 3, forty arable crop farmers were randomly selected from forty separate households of each village. From each household, one farmer aged 18 years and above was selected. A household where there was no farmer with the required attribute was substituted with another household until the intended number of respondents was reached. This procedure gave a total of 120 farmers. Also, in each of the same selected villages, 40 Sukuma herdsmen were randomly selected, using the same procedure, thus giving a total of 120 herdsmen. All these respondents were randomly selected from subvillage rosters of each of the three selected villages. A total of 252 respondents comprising 120 farmers and 120 agropastoralists including 6 government officials and 6 key informants who were purposively selected were interviewed for the study.  Figure 2 shows the location of the selected study villages.

Such stratified random sampling approaches, using agroecological zones as strata, have been usefully implemented among the Sukuma study population in Katavi [39–41]. Additionally, Snowball sampling that facilitates the identification of “key informants” was also employed for soliciting qualitative data.

### 3.5. Data Collection Techniques and Instrumentation

The approach employed by this study for data collection included both quantitative and qualitative methods. Although the positivists support the quantitative approaches on the ground that the world is composed of observable and measurable facts, the interpretivists, in contrast, support the qualitative methods on the ground that the world is in reality socially constructed, complex, and always in the process of change. This study, therefore, used both qualitative and quantitative approaches since they provide different perspectives and usually complement one another.

Quantitative primary data for this study were obtained from a field survey using a self-administered structured questionnaire. Respondents were interviewed at their new residences in the study area. The questionnaire had seven sections, each achieving particular predetermined objectives of the study. The sections comprising the questionnaire included the socioeconomic characteristics of the respondents, factors for the incursion into frontier landscapes, causes of conflicts, capabilities of local reconciliatory institutions, and knowledge and effectiveness of policies that regulate the use of land and water sources by the crop farmers and agropastoralists. Interview method was employed especially where nonliterate respondents who cannot complete the questionnaire themselves were encountered.

The administration of the questionnaire was complimented by qualitative data collection. A robust, in-depth interview of three separate key informants drawn from opinion leaders, village leaders, and outstanding men and women in the society was carried out in each study site. Key informants are respondents who are particularly knowledgeable and have deep insights that are helpful in understanding what happens [42]. Key informant interviews were carried out primarily to establish the historical evolution of the study settlements, the incursion of the Sukuma agropastoralists in frontier settlements, local norms and rules that guide settling, and the effectiveness of these local rules in guiding frontier areas' protection. An in-depth interview with village government leaders was meant to provide a ground to make inferences on policy issues and perceptions of the institutions they represent on planning and management of ethnic clashes occurring in frontier settlements. Two focus group discussions consisting of homogeneous participants, such as agropastoralists alone or their counterparts, were conducted at each study site to facilitate the collection of information and opinions on the causational factors that lead to conflicts and the possible solutions to such problems. Focus group discussions were held to explore variations in views of residents and their expressions of their environment.

Both individual in-depth interviews and focus group discussions were audiorecorded, transcribed, and analyzed. Data collection from study settlements was systematically administered from one study site to another in the period between August and December 2016.

### 3.6. Analytic Techniques

The analysis was based on a combined quantitative-qualitative approach. It was divided into 4 sections. The first section asked questions on socioeconomic-cum-demographic characteristics of respondents. Most of these questions were dichotomous in nature. The main characteristics inquired from agropastoralists and farmers included respondents' age, gender, marital status, level of education, occupation, herd size, household size, duration of residence, and land size. The analysis emanating from these characteristics helped to determine whether demographic factors trigger the conflicts in question.

A Logit Regression Model was run to measure the effects of socioeconomic factors as independent variables on natural resource-use conflicts as a dependent variable. The use of log odds ratio in logistic regression provided a simpler description of the probability relationship of the variables and the outcome. The model was used to estimate the probability of occurrence and nonoccurrence of resource-use conflicts in the frontier areas in Tabora Region. The binary dependent variable used was the existence of resource-use conflicts with the value of ONE if the response is YES and ZERO if the response is NO. In this study, education level, marital status, age, duration of residence, farm size, distance from home to resource base, household size, and gender were considered as independent variables to influence the existence of natural resource-use conflicts in the study area. A linear combination of these independent variables was established for prediction purposes. The following logistic regression model from [43] was used:

(1) $$\begin{array}{c} Y_{i}=\frac{1}{1+e^{-z}}, \end{array}$$

where *Y*_*i*_ is the *i*th observation value (score) of the dependent variable representing a linear combination of the independent variables underlying resource-use conflicts in the study area, which stand for a nonstandardized logistic regression equation. This was then used for prediction purposes. *Y*_*i*_ is a binary variable with a value of 1 if the respondent reported existence of resource-use conflicts in the study area and 0 if otherwise. *e* is natural logarithm equal to 2.718:

(2) $$\begin{array}{c} Y_{i}=\beta_{0}+\beta_{1}X_{1}+\beta_{2}X_{2}+\beta_{3}X_{3}+\cdots +\beta_{n}X_{n}+\varepsilon_{i}. \end{array}$$

*Y*_*i*_ is summation of independent variables. *X*_1_ to *X*_*n*_ are independent variables (education level, marital status, age, gender, duration of residence, farm size, herd size, distance from homestead to the resource base, and household size), *β*_0_ is constant term of the model without the independent variables, *β*_1_–*β*_*n*_ are independent variable coefficients showing the marginal effects (negative or positive) of the unit change in the independent variables on the dependent variable and these were used in developing prediction equations on the resource-use conflicts, *ε*_*i*_ is random error term, *i* = 1,2, 3,4,…, *N* (total number of respondents) is sample size (i.e., 252 for the purpose of this study), and *n* is total number of independent variables (*n* = 9). From the above, the independent variables in the model are elaborated as follows: *X*_1_ is level of education of the respondent (including years of schooling). It is assumed that increase in education level reduces the incidences of resource-use conflicts because educated people have more options to meet their livelihoods and reduce the chances of occurrence of conflicts in the study area. *X*_2_ is marital status of the respondent. There is a high probability of occurrence of resources-use conflicts with the increase in the number of married heads of households due to increased responsibilities of heads of households to meet household demands from different resources. *X*_3_ is age of the respondent in years. It is assumed that increase in age of the respondent reduces the incidences of resource-use conflicts because older persons are usually assumed to have accumulated enough resources to meet their livelihoods. Also, older people are assumed to have much wisdom related to resource use and in resolving resources-use conflicts through reconciliation committees. On the other hand, the increase in the number of young members in the community increases the chances of intergenerational conflicts in a given area because they want to accumulate enough resources for their households. *X*_4_ is duration of residence (years).

It is assumed that the more time a person stays in a particular area, the less the incidences in resources-use conflicts. This is because an individual who has stayed in a particular place for a long time is assumed to have owned enough land resources to meet his/her livelihoods than an immigrant to the area. *X*_5_ is household size. It is assumed that by increasing the number of members in a household, the resources-use conflicts also tend to increase due to an increased demand per individual. *X*_6_ is farm size (ha). It is assumed to have fewer incidences of resources-use conflicts when individuals acquire enough land because they are capable of allocating it for different uses. Land scarcity is assumed to have higher chances of resources-use conflicts. *X*_7_ is average distance (km) from homesteads to the resource base and the market. It is assumed that the closer the respondent's homestead is to the resource base and the market, the more likely the occurrence of resource-use conflicts is to be due to closeness to the resource base hence more frequent visits to exploit the resources. By increasing the distance from the resource base to homesteads, the incidences of resources-use conflicts in a given area are reduced. *X*_8_ is gender of respondents. Gender dimension reflects a clear division of labor at the household level as most African females do most of the household chores such as cooking, taking care of children, and housekeeping while males go out to search for opportunities to improve household welfare. On the other hand, gender as a social relation has a profound influence on the role men and women play in the management and conservation of natural resources. It was hypothesized that men visit forests and water points more frequently than women while searching for opportunities. Thus, in so doing, men are considered to bring more impact on the forests than what women can do based on the acquired field knowledge. *X*_9_ is herd size. The greater the size of the herd, the bigger the grazed area of the farm.

To test whether the regression coefficients are significantly different from zero, the Wald statistic that asymptotically follows a Chi-square distribution in large samples [44, 45] was used. The Wald statistic is distributed as Chi-square with a degree of freedom (df) equal to the constrained parameters (*r*) with a single parameter; the Wald statistic is simply the square of the *t*-ratio. The odds ratios represented by exp⁡(*β*) from logistic regression analysis were used in explaining the likely occurrence or nonoccurrence of resource-use conflicts in a study area under specified socioeconomic factors. Equation (1) was used to predict the probability of increasing resource-use conflicts under a given linear combination of independent variables (*Y*_*i*_). To evaluate the goodness of fit of the regression model to the data, the model Chi-square as suggested by [43] was used. By applying the Chi-square test, the significance level of the model was established at 5 percent probability level. Both nonstandardized equation (see (1)) and standardized equations (see (2) and (3)) using logistic regression coefficients (*β*) and exponential coefficients (exp⁡(*β*)) equivalent to beta weights (*b*^*∗*^), respectively, were developed.

(3) Prob  (Occurrence  of  resource  use  conflicts)=Prob  (Event)=ezi1+ezi

(4) The  Prob  (No  occurrence  of  resource  use  conflicts)=Prob  (No  event)=1−Prob  (Event)=1−ezi1+ezi.

Nonstandardized figures (*β*) are used in predictions of phenomena, whereas standardized figures (exp⁡(*β*)) are used to assess the relative effect of each predictor on the change in odds ratios. Thus standardized figures [exp⁡(*β*)] were used to explain the phenomena under the study.

It should be recognized that interpretation of parameters in logistic regression is not as straightforward and easy as it is the case with Ordinary Least Square (OLS) methods [43, 46]. For proper interpretation of results from a logistic regression model (LRM) the following was done: (i) the Wald statistics values were tested to see whether the effect of a particular independent variable was statistically significant, (ii) the sign of the effect (*β*) was tested too to see whether the independent variable increased or decreased the probability of success (in this case occurrence of resource-use conflicts in the study area), (iii) the relative magnitudes of the similarly measured variables were calculated, to determine which of the independent variables were examined to see which of the dependent variables seem to have greater impact on occurrence of resource-use conflicts in the study area; (iv) also the value of the exp⁡(*β*) was tested to see how much a unit increase in *X*_*n*_ changes the odds ratios of success (keeping in mind that the odds of success are not the same as the probability of success), and (v) different values for independent variables (*X*_*n*_) were substituted in the equation to see how changes in the value of a particular independent variable affect the probability of success or failure.

In section 2, agropastoralists were asked to free-list the factors responsible for migration into frontier landscapes. Since some farmers might have also moved to the frontier landscapes from another location, the independent (predictors) variables for this study included aspects of both groups. Respondents were asked, “What motives drove your out-migration from your settlement of origin?” Responses formed a basis for the variable “Out-migration.” Migration causation factors or reasons were evaluated by the use of a five-point Likert scale: “strongly disagree,” “disagree,” “neutral,” “agree,” and “strongly agree,” of which they were dichotomized for analysis. The analysis was presented in terms of adjusted odds ratios. Adjusted odds ratios and 95 percent confidence intervals were calculated by using logistic regression to adjust for potential confounders identified in bivariate analyses. Logistic regression examines the influence of various factors on dichotomous outcomes by estimating the probability of the event's occurrence. The main focus here was based on an individual or household level migration of which the outcome variable was measured as a count of migratory trips an individual has undertaken or a count of migrants in a household to the time of this survey. Factors or reasons associated with the migration could be demographic, social, economic, political, or environmental characteristics of individuals or households.

Both groups of respondents with rival interests were in section 3 asked another question, “What do you think could be the causes leading to farmer-herder conflicts?” On the basis of this main question, the predictor causes were derived from environmental, social, and political areas. The inferential statistical analysis was done using logistic regression. The logit is the natural logarithm (ln) of odds of *Y* [45]. The logit regression model is explicitly specified as

(5) Y=β0+β1X1+β2X2+β3X3+β4X4+β5X5+β6X6+β7X7+β8X8+β9X9+εi,

where *Y* is interpersonal conflict (any conflict mentioned = 1; otherwise 0), *X*_1_ is age of head of household, *X*_2_ is gender of head of household, *X*_3_ is marital status, *X*_4_ is educational level, *X*_5_ is farm size, *X*_6_ is household size, *X*_7_ is herd size, *X*_8_ is distance to resource base, *X*_9_ is duration of residence, *β*_0_ is constant, *β*_1_–*β*_9_ are regression coefficients, and *ε*_*i*_ is error term.

In section 4, agropastoralists and their counterparts were asked about their knowledge of Tanzania's policies and the effectiveness of local institutions on conflict resolutions. Logistic regression was employed for the prediction of performances of institutions supported by results from focus group discussions.

Overall, analysis of quantitative data employed both descriptive and inferential statistics. It was done with the help of Statistical Package for Social Sciences (SPSS) version 17 with a cut-off point set at *p* = .05 level of significance.

Qualitative data were gathered using Focus Group Discussion. Focus groups with eight members each were convened in each group. Three types of groups were chosen based on shared characteristics in experiences to exposure of environmental resources scarcity and their likely effects on livelihoods: community/civic leaders, farmers, and herders. The gathered data were analyzed using thematic analysis facilitated by MAXQDA.

### 3.7. Pretesting of the Questionnaire

This test was carried out among 30 respondents who were not included in the research sample. Experts assessed the questionnaire and a pilot test was conducted among the Sukuma agropastoralists and crop farmers' communities at Utimule, Sikonge District. Items that were found ambiguous were removed, and the wording of the questions was restructured to make them clearer.

### 3.8. Validity and Reliability of the Data

Reliability of the questionnaire was checked for internal consistency. This data collection instrument was subjected to a split half method which assesses the internal consistency. It measures the extent to which all sections of the questionnaire contribute equally to what is being measured. The method required splitting the contents of the questionnaire into two halves: either odd/even number or first/second half (usually the odd-numbered items are scored separately from the even-numbered items). This was followed by correlating the total score of one-half with the total score of the other, using the Pearson product-moment correlation formula:

(6) $$\begin{array}{c} r=\frac{\sum \left(X-\overset{-}{X}\right)\sum \left(Y-\overset{-}{Y}\right)}{\sqrt{{\sum \left(X-\overset{-}{X}\right)}^{2}}\sqrt{{\sum \left(Y-\overset{-}{Y}\right)}^{2}}}, \end{array}$$

where *X* means average (array 1) and *Y* is sample and average (array 2).

The outcome coefficient is an estimate of the half-test reliability of the entire test (i.e., the reliability of the odd-numbered elements, or the even-numbered elements, but not both merged). This statistical test consists of looking at the correlation coefficient. If it is sufficiently high, then the questionnaire is considered to be reliable. One problem with the split-half reliability coefficient is that since only half the number of items is used, the reliability coefficient value is therefore reduced. To adjust the reliability of the half test, this study applied the Spearman-Brown prophecy correction formula.

(7) $$\begin{array}{c} \text{Reliability}=\frac{2\times r\;\text{half test}}{1+r\;\text{half-test}} \end{array}$$

If the result of the correction is high enough, it shows that the test is quite reliable. The Spearman-Brown prophecy coefficient for this study was 0.764 which is reasonably acceptable.

The questionnaire was further subjected to test-retest reliability by giving the same tool to the same respondents on two separate occasions and then correlating the scores. This testing ascertains the stability and reliability of an instrument over time. The test-retest reliability of this questionnaire (which comprises categorical data) was analyzed using the kappa statistic [47]. This statistic is a measure of a test on whether agreement exceeds chance levels: *k* = (*p*_*o*_ − *p*_*e*_)/(1 − *p*_*e*_), where *p*_*o*_ is the proportion of an observed agreement and *p*_*e*_ is the proportion of an agreement expected by chance, the joint probabilities of the marginal proportions. In consistence with [48], this study defines observed and expected agreement as *p*_*o*_ = (*a* + *d*)/*N* and *p*_*e*_ = (*f*_1_ *g*_1_ + *f*_2_ *g*_2_)/*N*^2^, where *a* and *d* represent positive and negative agreements, respectively, in a 2 × 2 table presented in Table 2.

Kappa values greater than 0.75 were regarded excellent; values between 0.40 and 0.75 are fair to good and values less than 0.40 represent poor agreement beyond chance [49]. This study yielded a Cohen's Kappa statistic of 0.737 which is adequate.

As regards validity, the questionnaire was subjected to content validity and face validity to assess the extent to which the instrument measures what will be designed to measure. Content validity here means the degree to which items on a questionnaire adequately cover the construct being studied. Increasing the number of various measures in a study leads to increasing construct validity provided that the measures are measuring the same construct. Face validity, on the other hand, was assessed by looking at the measure whether it measures what it is supposed to measure. Evidence of validity was found in the* content*,* response process*,* relationships to other variables*, and* consequences*.

### 3.9. Ethical Consideration

Prior to interviewing households, meetings were held with village officials to solicit their consent and obtain household lists from which households for the study were picked randomly.

## 4. Results and Discussion

### 4.1. Socioeconomic Characteristics of the Respondents

The summary of personal characteristics of respondents is presented in Table 3. Although 260 respondents were involved in the research, 252 questionnaires were used in data analysis as the remaining seven were either not returned or poorly completed, giving a response rate of 97 percent.

Results of the socioeconomic characteristics of the respondents are presented in Table 3. Most of the crop farmers (52.38 percent) as well as the agropastoralists (47.62 percent) fall in the age range 31–50 years while only a few of the respondents were above seventy years of age. The average age of farmers was 44 years. Also it can be seen that majority of the respondents (both crop farmers and agropastoralists) were males. This explains the fact that most of those who practice crop farming or are engaged in grazing of animals and farming are mostly matured males. However, in reality, it is the women who are engaged in crop farming, but when there is a conflict, it is the men who carry out the attack or go to courts. For the counterparts (agropastoralists), it is the men who are engaged in grazing of livestock. Although Table 3 shows the preponderance of male-headed households in the study area, about 26 percent of respondents were women household-heads.

The table also shows that 91.27 percent of crop farmers and 83.33 percent of agropastoralists have a family size of between 1 and 10 persons. These are peculiar situations in rural areas as most of these families lack the basic resources for development and consider land or cattle their only source of livelihood. Also, most of these farmers and agropastoralists believed that it is better to have more children who will work on the farm or help in grazing the herds than hiring external labor. Most of the crop farmers had at least a primary school education (71.43 percent) while 15.08 percent had no formal education. On the contrary, the majority of the agropastoralists (30.16 percent) had no formal education. This goes to show that education among the agropastoralists is not considered a priority because they are known for their seminomadic life style which makes them migrate from place to place with their* lubaga* (grazing practice of the Sukuma herdsmen) herds during the dry seasons. The proportion of agropastoralists who had moved into the frontier areas were more (53.17 percent) than the proportion of crop farmers (29.37 percent) who comparatively had a longer period of residence (15–20 years). Furthermore, 35.65 percent of farmers had a farm size of less than one hectare while only 9.72 percent of the farmers had a farm size of above five hectares. This goes to confirm that land holdings in the rural areas are usually small and are obtained mostly through inheritance. This shows the preponderance of small-scale farming among respondents as noted in [50]. On the other hand, 42.17 percent of grazers have cattle herds of 50 and below while 2.17 percent had a herd size of above 300 cattle. The number of cattle a man has is considered as a sign of wealth. Therefore, those with a heard of 50 or below are considered poor among the agropastoralists.

### 4.2. Influence of Demographic Variables on Land and Freshwater Scarcity Induced Conflicts

Logistic regression was performed to determine the influence of demographic variables on land and freshwater scarcity induced conflicts among the conflict actors (crop farmers and agropastoralists). Results from the study (see Table 4) show that demographic variables like age, gender, marital status, the level of education, income level, and land allocated for crop production are not significantly related to detrimental environmental destruction and rampant conflicts between the farmers and pastoralists except household size, residence, and herd size.

Sex of the respondents has a positive estimated logit coefficient (*β* = .767) and odds ratios of 2.153. This implies that for every increase in one person the perception on odds of an event to occur is not statistically significant (*p* = .121), increasing by a factor 2.153. Sex impact on the natural resources-use conflicts was not statistically significant, which means that resource-use conflicts would occur irrespective of the sex of the resource user. These findings concur with studies conducted in [51] on assessing knowledge of stakeholders regarding the concession process in the forest management.

The negative regression coefficient (*β* = −.162) in education level implies that a decrease in the education level of respondents increases the odds ratios of resources-use conflicts by a factor of .851. This is because a decrease in education level is normally associated with a decrease in understanding of the broad benefits accrued from the conservation of natural resources. As discernible from Table 1, the majority of the respondents from the rivalry groups have attained primary education only. It, therefore, goes to say that decreased level of education also decreases options of respondents to meet their livelihoods. Limited postformal education among rural population causes “inadequate” awareness of environmental issues.

Marital status of respondents has a positive regression coefficient (*β* = .163). This implies that marital status causes heads of most households to look for more household basic needs. However marital status was not statistically significant (*p* = .803), indicating that the sampled households had enough land thereby not warranting conflicts with others. This finding coincides with author of [52] who in his study in Northern Nigeria also found that demographic factors did not have a robust relationship with the conflict between farmers and pastoralists due to freshwater scarcity.

Age of a respondent has a positive regression coefficient (*β* = −.012) and an odds ratio of 1.038. This factor was found not statistically significant (*p* = .183) on the natural resources-use conflicts in the study area.

The land allocated for crop production had a negative estimated logit coefficient (*β* = −.384) implying that a decrease in farm size increases the chances of resource-use conflicts by a factor of .681. Plattaeu [53] explained that when land acquires a scarcity value, landholders begin to feel uncertain about the strength of their customary rights, and disputes over ownership of land, inheritance, and land boundaries tend to multiply. Crop production in frontier landscapes has received a high priority followed by livestock keeping and has a high influence on the occurrence of resource-use conflicts. Land allocated for crop production was not statistically significant (*p* = .075). However, results in Table 4 indicate a Wald statistic of nonzero value (3.172) implying that there are interactions between the independent and dependent variables. According to [43, 46], nonzero values for the Wald statistics indicate the presence of relationships between the explanatory variables.

Farm income, influencing farm capital, could determine farm size and, consequently, the farmer's conflict experiences. This predictor yielded a negative (−.052) regression coefficient with an odds ratio of .924 implying that low income earned from a trespassed farm may lead to conflicts with agropastoralists. Being insignificant (*p* = .949), this variable is consistent with the submission of [54] that low income could be associated with conflict in developing countries.

The more time a person stays in a particular area, the less the incidences in resources-use conflicts. The results in the logistic regression (Table 4) indicate that duration of residence of respondents has a negative regression coefficient (*β* = −.052). This implies that an increase in years of duration of residence of a respondent reduces the odds ratios of natural resources-use conflicts by a factor of .949. The logistic regression results indicated that the impact of duration of residence on natural resources-use conflicts was statistically insignificant (*p* = .121).

Household size had a positive estimated logit coefficient (*β* = .042), which implies that by increasing the household size the odds of resource-use conflicts are increased significantly (*p* = .011). Herd size whose positive regression coefficient (*β* = 1.237) was significant at *p* = .019 increases the likelihood of resource-use conflict by a factor of 3.444. The significance of herd size in influencing the conflicts among the farmers and agropastoralists might be because increasing herd size requires more pastureland and water and thus encourages more encroachment and destruction to the farm and its crops and water sources.

### 4.3. Factors Driving Migration into Frontier Landscapes

A logit regression analysis was conducted to predict the influence of various factors driving migrants' decisions to settle in frontier landscapes.  Table 5 summarizes results of the predictors in the study area. The model predicted correctly the relationship at 90.4 percent and significantly at *p* < .05.

Predictors with positive influence are perceived degradation of rangeland, land for agriculture, grazing land, rainfall, family size, and business. Perceived deterioration of grazing land significantly (*p* = .004) increased the likelihood of migrating to frontier areas by a factor of 4.157. The perceived deterioration of grazing land was used as a proxy to the scarcity of pasture and water in rangelands of places of origin. This could be one of the reasons for agropastoralists to engage in the seasonal movement of their herds to alternative grazing lands in frontier forests. The results also show that availability of virgin land for agriculture in frontier areas significantly (*p* = .002) increased the likelihood of migration into such new landscapes in Tabora Region by a factor of .295. This has led to increasing grabbing of land by new settlers. Grazing land also significantly (*p* = .000) increased the likelihood of migration into frontier lands by a factor of 28.292. Rainfall has a positive influence on the migration of both agropastoralists and crop farmers with a positive estimated logit coefficient (*β* = 2.398) and a Wald ratio of 4.314. The relationship is significant at *p* = .038. This suggests that ample rainfall in forests is likely to increase human and livestock movement into such areas.

Local community kin ties also have a strong positive relationship (*β* = 1.091) with movement into frontier areas with a Wald ratio of 1.769. The results suggest that the increase in some local community kin ties is likely to increase the likelihood of migration into frontier areas by both agropastoralists and crop farmers alike. Although the relationship is not significant at *p* = .183, the explanation for this is that since both parties have social networks created by kins, this variable becomes a driving factor for them to move to such areas. Family size is also positively related to migration into frontier landscapes (*β* = .013) with a Wald ratio of 2.413 and so does business with a positive regression coefficient (*β* = .510) and a Wald ratio of .517. The Wald criterion demonstrated that family size and business made a significant contribution to prediction (*p* = .013 and *p* = .032, resp.). The plausible explanation is that agropastoralists, particularly the youth, do not engage in urban area migration. This owes to the fact that because frontier areas are well connected to the market system, this offers opportunities for generating incomes through trading cattle in nearby urban centers. Consequently, the youth have incentives to remain in the area where they engage in herding and transhumant livestock movements.

### 4.4. Determining Factors for Conflicts

The results in Table 6 show the estimated coefficients of the variables contributing to resource-use conflicts in the study area.

The model parameters predicted correctly at 92.2 percent and significantly at *p* < .05. The log likelihood function of 26.405 indicated a high fit between the model data. Whereas the Nagelkerke *R* square of .846 suggested that the variables in the model account to about 84.6 percent of the observed variation in the variables under study.

Age of head of household in years: for every unit increase in age, the log odds of conflict being resolved in the past increase by .72. This factor was found not statistically significant (*p* = .272) on the natural resources-use conflicts in the study area. This could be that young generations from the sampled households had acquired enough knowledge from their elders on how to protect and manage their farm resources thereby avoiding unnecessary conflicts.

Marital status of respondents has a positive regression coefficient of .045. This implies that marital status causes heads of most households to look for more household basic needs. However marital status was not statistically significant (*p* = .250), indicating that the sampled households had enough land to meet their livelihoods thereby not warranting conflicts with others. As regards sex of respondents, its impact on the natural resources-uses conflicts was not statistically significant (*p* = .217), which means that resource-use conflicts would occur irrespective of the sex of the resource user. The negative coefficient (−1.215) in education level implies that a decrease in the education level of respondents increases the odds ratios of resources-use conflicts by a factor of .297. This is because decreased level of education also decreases options of respondents to meet their livelihoods. Limited postformal education among rural population causes “inadequate” awareness of environmental issues.

The results show that the increasing herd size of individual pastoralists with a regression coefficient (*β* = 4.276) contributed significantly (*p* = 0.001) to the likelihood of conflicts with farmers by a factor of 7.197. The plausible explanation of this is that when a household increases the herd size, demand for grazing land increases. In turn, this necessitates high herd mobility which increases the likelihood to trespass into farmers' villages thus causing crop damage which leads to conflicts with farmers. This is congruent with [55] in which it was stated that the most frequent cause of such conflict is the destruction of crops by cattle. These cattle enter the farm to feed on the foliage of crop even in the presence of the herdsmen who pretend not to notice such destruction. This supports [56] in which it was averred that, in the preharvest period, cattle frequently destroy or eat ripened crops as they are led from the field to their kraals.

As regards farm size, the land allocated for crop production had a negative regression coefficient of −1.734. It was observed that a unit decrease in farm size increases the odds of resource-use conflicts by a factor of .176. The explanation for this is that farmers with smaller farms are more likely to wage conflicts with herders when livestock trespass and destroy crops in farms. This variable has also been reported in [24, 57, 58], in which it was argued that land shortage both for crop cultivation and for livestock production is one of the driving forces underlying these conflicts. These conflicts have forced users to encroach forest areas to fulfil individuals' interests.

Household size is positively related to resources-use conflicts, with a positive regression coefficient (*β* = .958) and a Wald ratio of 3.133 which implies that by increasing the household size the odds of resource-use conflicts are increased significantly (*p* = .017). The conflicts between herdsmen and crop farmers are escalated when the crops used by farmers to feed their large household members are threatened.

As is discernible from Table 6, distance to resource base was found to be insignificant (*p* = .067) with a negative regression correlation (*β* = −.812) reducing the probability of the occurrence of conflicts. This negative relationship tells us that the larger the distance, the lesser the tendency of conflicts. The possible justification could be households who are closer to their farms make a closer follow-up against destruction by herds of livestock as opposed to those with distant farms.

As regards duration of residence, the more time a person stays in a particular area, the less the incidences in resources-use conflicts. The results in the logistic regression indicated that duration of residence of respondents has a negative regression coefficient of −1.401. This implies that an increase in years of duration of residence of respondent reduces the odds ratios of natural resources-use conflicts by a factor of .246. The impact of duration of residence on natural resources-use conflicts was statistically insignificant (*p* = .146).

### 4.5. Knowledge of Tanzania's Policies and the Effectiveness of Local Institutions

Respondents were finally asked to evaluate the effectiveness of the institutions involved in managing farmer-herder conflicts in the study area.  Table 7 presents the efficiency and performance of the stakeholders in conflict resolutions based on logistic regression.

Institutions with positive influence include village land tribunals, village environmental committees, ward land tribunals, and district housing and land tribunals, while institutions with negative influence are rangers, village leaders, and the police. Rangers have been perceived to be ineffective in mediating conflicts. This is consistent with the findings of [59] in which it was reported that crop damage by wildlife brings enmity between wildlife authorities and crop cultivators because there is no compensation for the damage to crops caused by wild animals. The highly significant positive relationship between ward land tribunals (*p* = .00) and management of communal grazing lands in the study areas can be explained by the fact that they are efficient and this may be a result of the inclusion of members from the village environmental committees.

It can also be inferred from the table that the village land tribunals and the ward land tribunals together with the village environmental committees and district housing and land tribunals are the most successful institutions that can manage conflicts, while the police and rangers are regarded as unsuccessful. This is probably due to the following reasons: people live and respect their leaders, while the police, rangers, and local government officials are perceived to be corrupt and make unequal treatment when the conflicts occur. This is consistent with the findings of [60, 61] in which it was argued that unless local communities adjacent to protected areas are involved in comanagement of natural resources, resource-use conflicts will remain inevitable in Tanzania.

The intervention of the district housing and land tribunals made a significant contribution to prediction (*p* = .00) as they can significantly increase the likelihood of cooperation in the management of common rangelands by a factor of 2.951. The explanation for these results is the fact that the major role played by the district housing and land tribunals, besides settling disputes, fosters consultations with host communities prior to the resettlement of the agropastoralists from other areas facing environmental stress. Without such measures, the host communities are likely to feel that they have been invaded by agropastoralists with the assistance of the government.

The focus group discussions further corroborated the above findings and further illuminated the factors the rivalry groups described as important in their coexistence. The agropastoralists spoke with less enthusiasm about their education and gave little evidence of knowledge of relevant land policies. One group of middle-aged agropastoralists, all of whom being Sukuma by ethnicity, displayed little knowledge of the land policy in Tanzania, agreeing with one of their participants who said: “We don't know the land policy. Additionally, we face problems of access to and use of land posed by the prevailing land tenure since it impedes fair resource management.” They admitted sometimes trespassing on some crop farms because as one participant said “in some crop farms, we don't get convenient passages for our herds when taking them to open grazing lands and water points. This is because the farms are too fragmented and close to one another.” They spoke strongly about their loyalty as citizens. One participant pointed out that “we endorse the mediation role played by the village environmental committees as well as the ward land tribunals.” However, this support was not directed to the police and rangers as they were blamed for often taking bribes from them. The focus group interviews supported the conclusion that nomadic education breeds economic sustainability of land resources-users and empowers them to take responsibility for their development.

The crop farmers' focus group discussions provided strong evidence to support the importance of coexistence when one participant said “both farming and pastoral groups must learn to respect each other's rights in their interactions. The farmers should avoid encroaching on the stock routes mapped out for the pastoralists, while the pastoralists on the other hand must avoid the indiscriminate destruction of crops in the farms.” In a different group, an elder farmer spoke disparagingly about the recurring conflicts: “many farmers often lose part or whole of their crops. These incidences reduce yields which are translated into low income on the part of us who take farming as a major occupation. They tend to negatively affect our savings, credit repayment ability, and food security. The losses contribute to the prevalence of poverty among farmers.” When probed for their recommendations, one participant said “conflicts between pastoralists and crop farmers must be reduced significantly through participatory approaches pursued by the village land tribunals. These tribunals usually demarcate lands for each party. This process is good because it involves all stakeholders and stimulates economic growth leading to poverty-reduction.”

## 5. Conclusion

From the study, it was discovered that there are recurrent clashes of interests between the host farming communities and the agropastoralists along the frontier landscapes. The study has revealed that natural resources are limited to accommodate all needs of the rapidly growing population in Tabora Region. The scarcity of resources forces the two rivalry communities to compete for resources-uses thus resulting into conflicts. The predicted underlying causes of such conflicts were found to be basically due to socioeconomic factors which include a low level of education, household size, farm size, and herd size. Institutional factors which positively predicted conflicts included inadequate land tenure arrangements and poor participation of local communities in the management of natural resources programs. The study concludes that the existing natural resources conflicts in the study area were basically due to socioeconomic factors where the weak institutions made local communities compete for water and land resources in the area without successfully managing them. Strengthening of the existing local institutions has been identified as a means to reduce resources-use conflicts and bring about sustainable management of natural resources in the region. The crop-livestock linkage is suggested to be one of the key sources of sustainable natural resources-use conflicts management in the study area. To bring about fruitful results, this recommendation calls for concerted efforts to educate farmers and livestock keepers on crop-livestock linkages so that they may improve their levels of productivity and get away with agricultural extensification into frontier landscapes. There is as well a need for population growth control and the creation of awareness of land-use regulations among farmers and herdsmen. This step can be accomplished through education and strengthening of favorable national policies. This would not only lead to enhancing mutual understanding and tolerance but also lead to the creation of a better opportunity for awareness of realistic coping strategies.

## Figures and Tables

**Table 1:** Distribution of study villages in Tabora Region.

District	Ward	Village
Nzega	Karitu	Bugembe
Igunga	Simbo	Tambalale
Uyui	Kigwa	Kigwa
Urambo	Ichemba	Ichemba
Sikonge	Ngoywa	Mabangwe
Kaliua	Kangeme	Kangeme
Tabora Urban	n.a.	n.a.

n.a. = not applicable. Source: Regional Administrative Office, Tabora, 2016.

**Table 2:** 

		Round 2		Total
		Yes	No	
Round 1	Yes	*a*	*b*	*g*_1_
	No	*c*	*d*	*g*_2_
		*f*_1_	*f*_2_	*N*

**Table 3:** Socioeconomic characteristics of respondents (*n* = 252).

Variable	Agropastoralists (frequency)	Percentage	Crop farmers (frequency)	Percentage
*Gender*				
Males	98	77.78	89	70.63
Females	28	22.22	37	29.37
Total	126	100.00	126	100.00
*Ages (years)*				
15–30	38	30.16	52	41.27
31–50	65	51.59	64	50.79
51–70	22	17.45	8	6.35
>70	1	0.80	2	1.59
Total	126	100.00	126	100.00
*Marital Status*				
Married	88	69.84	89	70.63
Never married	21	16.66	17	13.49
Divorced	7	5.55	5	3.97
Widowed	9	7.15	14	11.11
No response	1	0.80	1	0.80
Total	126	100.00	126	100.00
*Educational level*				
No formal education	38	30.16	19	15.08
Primary school	85	67.46	90	71.43
Secondary school	3	2.38	16	12.69
Tertiary education	0	0	1	0.80
Total	126	100.00	126	100.00
*Occupation*	120	47.62	132	52.38
*Household size*				
1–10	105	83.33	115	91.27
11–20	21	16.67	11	8.73
>30	—	—	—	—
Total	126	100.00	126	100.00
*Duration of residence *				
<15 yrs	67	53.17	37	29.37
15–20 yrs	39	30.96	55	44.65
>30 yrs	20	15.87	34	26.98
	126	100.00	126	100.00
*Herd size (number of animals)*			*Land size (ha)*	
<1	—	—	30.09	
3–4.99	—	—	53	24.54
>5	—	—	21	9.72
<50	97	42.17	—	—
51–100	65	28.26	—	—
101–200	39	16.96	—	—
201–300	24	10.44	—	—
>300	5	2.17	—	—

*Note*. Mean age = 44 years. Mean family size was 9; Mean farm size = 2.8 ha. Source: Tabora Agropastoral Mobility Survey, 2016.

**Table 4:** Results of logistic regression showing the predictive effect of demographic factors on conflicts over natural resource access.

Variables in the equation	Estimates					
	*β*	SE	Wald	df	*p*	exp⁡(*β*) Odds ratio
Sex	.767	.495	2.402	1	.121	2.153
Age	−.012	.011	1.822	1	.183	1.038
Education	−.162	.088	3.394	1	.065	.851
Marital status	.163	.632	.064	1	.803	1.173
Income level	−.052	.543	.011	1	.924	.949
Residence (0–10 years)	−.052	.024	5.901	1	.121	.949
Household size	.042	.033	2.713	1	.011^*∗*^	1.052
Land allocated for Crop production	−.384	.216	3.172	1	.075	.681
Herd size	1.237	.525	5.539	1	.019^*∗*^	3.444
Constant	.765	1.339	.327	1	.000	2.149

*Model summary*						
Number of observations = 252						
Overall percentage = 86.95						
Model *χ*^2^ = 22.69 at *p* < 0.05						
−2 log likelihood = 118.331						
Nagelkerke *R* squared = .743						

^*∗*^Significance at *p* < .05. Source: Tabora Agropastoral Mobility Survey, 2016.

**Table 5:** Logistic regression results predicting factors influencing migration of agropastoralists into frontier landscapes.

Variables in the equation	Estimates					
	*β*	SE	Wald	df	Sig.	exp⁡(*β*)
Perceived degradation of rangelands	1.425	.499	8.162	1	.004^*∗*^	4.157
Land for agriculture	−1.222	.0394	9.631	1	.002^*∗*^	.295
Grazing land	3.343	.832	16.121	1	.000^*∗*^	28.292
Family size	.013	.009	2.413	1	.013^*∗*^	1.014
Rainfall	2.398	1.154	4.314	1	.038^*∗*^	10.999
Business	.510	.710	.517	1	.032^*∗*^	1.666
Local community kin ties	1.091	.820	1.769	1	.183	2.976
Constant	−9.561	1.662	33.122	1	.000	.000

*Model summary*						
Number of observations = 252						
Overall percentage = 90.4						
Model *χ*^2^ = 79.13, at (*p* < .05)						
−2 log likelihood = 115.10						
Nagelkerke *R* squared = .754						

^*∗*^Significance at *p* < .05. Source: Tabora Agropastoral Mobility Survey, 2016.

**Table 6:** Results of logistic regression indicating factor estimates that determine resources-use conflicts.

Variables in the equation	Estimates					
	*β*	SE	Wald	df	Sig.	exp⁡(*β*)
Age of HH head	−.063	−1.299	.196	1	.272	0.72
Gender of HH head	1.465	1.187	1.522	1	.217	4.326
Marital Status	.045	.089	.693	1	.250	1.154
Educational level	−1.215	.619	3.852	1	.050^*∗*^	.297
Farm size	−1.734	1.261	1.891	1	.048^*∗*^	.176
Household size	.958	.541	3.133	1	.017^*∗*^	2.607
Herd size	4.276	1.201	12.673	1	.000^*∗*^	7.197
Distance to resource base	−.812	.532	2.330	1	.067	2.253
Duration of residence	−1.401	.762	3.379	1	.146	.246
Constant	14.469	4.153	12.140	1	.000	.000

*Model summary*						
Number of observations = 252						
Overall percentage = 92.20						
Model *χ*^2^ = 61.098						
−2 log likelihood = 26.405						
Nagelkerke *R* squared = .846						

^*∗*^Significant at *p* < .05 level. Source: Tabora Agropastoral Mobility Survey, 2016.

**Table 7:** Results of logistic regression showing perceptions on the effectiveness of conflict reconciliatory institutions.

Variables in the equation	Estimates					
	*B*	SE	Wald	df	*p*	Odds ratio
Rangers	.801	.522	2.323	1	.133	2.473
Village leaders	−.122	−.366	2.527	1	.012^*∗*^	.885
Village land tribunals	.962	.374	6.644	1	.014^*∗*^	2.612
Ward land tribunals	1.943	.362	29.661	1	.006^*∗*^	6.923
District housing and land tribunals	1.082	.312	11.951	1	.003^*∗*^	2.951
Village environmental committees	1.694	.283	35.774	1	.008^*∗*^	5.403
Police	−.912	.491	3.412	1	.043^*∗*^	4.582
Constant	.134	.366	.133	1	.715	1.143

*Model summary*						
Number of observations = 252						
−2 log Likelihood = 73.843						
Cox & Snell *R* Square = .149						
Nagelkerke *R* Square = .327						
Model *χ*^2^ = 26.687 (*p* < .05)						
Overall percentage = 92.1						

^*∗*^Statistically significant at .05 level of significance. Source: Tabora Agropastoral Mobility Survey, 2016.
